# Impact of combining vitamin C with radiation therapy in human breast cancer: does it matter?

**DOI:** 10.18632/oncotarget.28204

**Published:** 2022-02-22

**Authors:** Somayeh Khazaei, Linn Nilsson, Gabriel Adrian, Helga Tryggvadottir, Elise Konradsson, Signe Borgquist, Karolin Isaksson, Crister Ceberg, Helena Jernström

**Affiliations:** ^1^Division of Oncology, Clinical Sciences in Lund, Lund University and Skåne University Hospital, Lund, Sweden; ^2^Department of Hematology, Oncology and Radiation Physics, Skåne University Hospital, Lund, Sweden; ^3^Department of Medical Physics and Engineering, Växjö Central Hospital, Växjö, Sweden; ^4^Department of Research and Development, Region Kronoberg, Växjö, Sweden; ^5^Department of Clinical Sciences in Lund, Medical Radiation Physics, Lund University, Lund, Sweden; ^6^Department of Oncology, Aarhus University and Aarhus University Hospital, Aarhus, Denmark; ^7^Division of Surgery, Department of Clinical Sciences in Lund, Lund University, Lund, Sweden; ^8^Department of Surgery, Kristianstad Hospital, Kristianstad, Sweden

**Keywords:** human breast cancer, radiation therapy, vitamin C, *in vitro*, clinical outcome

## Abstract

Vitamin C may impact the efficiency of radiation therapy (RT) in breast cancer. The effects of RT alone or in combination with vitamin C in SKBR3, MDA-MB-231, and MCF7 cells were compared using clonogenic assay, proliferation assay (MTT), cell cycle analysis, and Western blot. Vitamin C use was assessed in 1803 breast cancer patients 2002–2017 in relation to clinicopathological features and recurrences after RT. Vitamin C combined with RT resulted in non-significant increases in colony formation and minor differences in cell cycle arrest and expression of studied proteins, compared to RT alone. Lower vitamin C doses alone or in combination with RT, resulted in higher proliferation with MTT than higher vitamin C doses in a cell line-dependent manner. Vitamin C use was associated with lower histological grade and BMI but not recurrence risk in RT-treated patients (LogRank *P* = 0.54). Vitamin C impacted RT efficiency differently depending on breast cancer subtype and vitamin C concentration. Lower doses of vitamin C, achievable with oral administration, might increase breast cancer cell proliferation and decrease radiosensitivity. Despite vitamin C users having less aggressive tumors than non-users, the recurrence risk in RT-treated patients was similar in vitamin C users and non-users.

## INTRODUCTION

Currently, complementary and alternative medicines and dietary supplements, including antioxidants such as vitamin C, are used by many breast cancer patients [1, 2]. The majority of breast cancer patients are treated with surgery, radiation therapy (RT), and often systemic therapy [3]. The efficacy of these treatments may be impacted by interactions with concomitant medications and dietary supplements, including vitamins [4]. Many supplements have antioxidant properties [5] that may affect chemotherapy and RT [6].

The majority of breast cancer patients receive RT as adjuvant treatment. Postoperative RT reduced both recurrence and breast cancer mortality [7–9]. RT acts on the cancer cells by causing single-stranded breaks and double-stranded breaks in the DNA, where a majority of the damages are due to radiation-induced highly reactive free radicals [10], leading to different types of stress responses, such as cell death, cell cycle arrest, and gene induction, involving multiple signaling pathways [11].

Since the main therapeutic effect of RT is through the production of reactive oxygen species, antioxidants may interfere with RT efficacy [12]. Taking (dietary) antioxidants, including vitamin C, concurrently with chemotherapy or RT, decreased the treatment’s effect; therefore, patients might be advised against it [6]. The risk for clinically significant interactions between vitamin C and RT needs further investigation.

Vitamin C at pharmacological concentrations in the blood might be a pro-drug for tissues delivery of the free radical H_2_O_2_ [13]. Therefore, the concentration of vitamin C might also play a role. However, clinical pharmacokinetics analyses show that pharmacologic concentrations of plasma vitamin C, from 0.3 to 15 mM, are achievable only by intravenous vitamin C administration. In contrast, plasma vitamin C concentrations from the maximum possible oral dose (physiological concentration) do not exceed 0.22 mM [13, 14]. The plasma concentration of vitamin C reaches about 50 μmol/l with 100 mg/day intake [15, 16], which is the recommended daily intake of vitamin C in some European countries [17]. The Swedish Food Agency recommends 75 mg/day for adults [18]. In patients, the half-life of vitamin C depends on the plasma concentration and route of administration [16]. There are only a few preclinical studies of the effects of physiological or pharmacological doses of vitamin C in combination with irradiation of cancer cells [19–23], and none have been performed in breast cancer cells.

Different breast cancer subtypes, as well as cell lines, show different levels of radiosensitivity [24, 25], for example, significantly improved overall survival after radiation was seen in patients with Estrogen receptor (ER)^+^ and Human Epithelial Growth Factor Receptor-2 (HER2)^–^ tumors, but not in patients with ER^–^ and HER2^+^ tumors. Furthermore, different breast cancer cell lines, including SKBR3 (HER2^+^), MDA-MB-231 (Triple-negative), and MCF7 (ER^+^), differ in their sensitivity to vitamin C, including vitamin C induced cell death [26].

The current study aims to elucidate how vitamin C treatment before or after irradiation impacts irradiation response in three different breast cancer cell lines. We hypothesize that treatment with vitamin C before or after irradiation interferes with the efficacy of irradiation in breast cancer in a cell line-dependent manner. Further, we aimed to investigate the frequency of vitamin C use in a cohort of breast cancer patients and whether vitamin C use was associated with clinicopathological features or outcomes after radiation treatment. Herein we report the impact of combining of vitamin C and irradiation on proliferation and survival rates of breast cancer cells, the induction of cell cycle arrest, and the proteins involved in these processes in a cell line dependent manner, as well as clinical data on vitamin C in breast cancer patients.

## RESULTS

### The sequential combination of vitamin C and irradiation showed no significant alteration compared to irradiation alone in three cell lines

**Figure 1:**
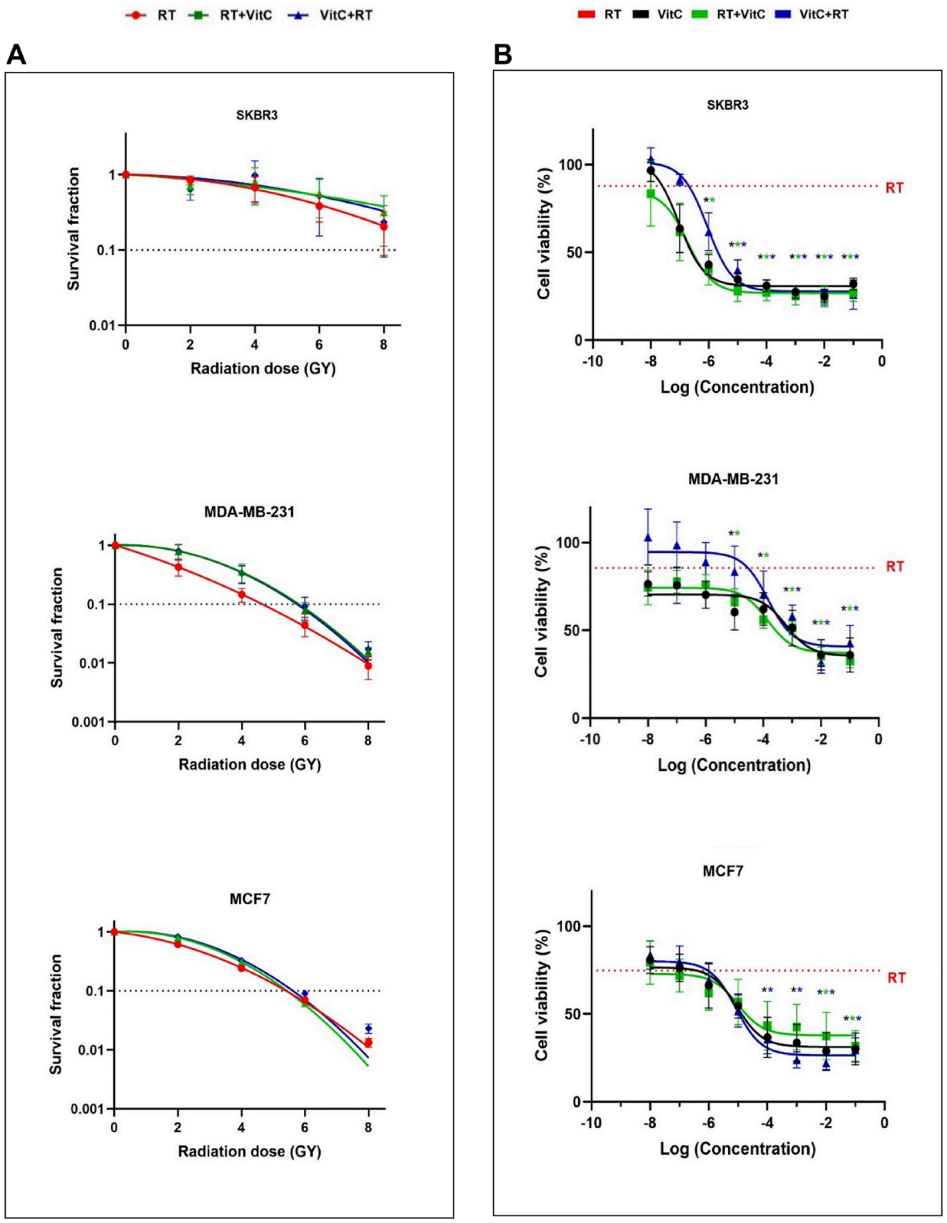
(**A**) Effect of VitC and/or different doses of irradiation on clonogenic survival of breast cancer cells. SKBR3, MDA-MB-231, and MCF7 cells were exposed to 2, 4, 6, and 8 Gy of irradiation in the presence or absence of 220 μM VitC. Colony formation in representative flasks of non-irradiated (CTRL) and 8 Gy irradiated cells alone or in combination with VitC is represented. The results are shown as the mean of three sets of experiments in triplicate samples (± SEM). Statistically significant differences compared to irradiated cells alone were calculated using Student’s t-test (^*^ *P* < 0.05). (**B**) Breast cancer cell viability after different doses of VitC and/or 8 Gy irradiation. SKBR3, MDA-MB-231, and MCF7 cells were treated with VitC before or after irradiation. Cell viability was determined by MTT assay. Values correspond to the mean (± SEM) of three independent experiments. Statistically significant differences compared to RT alone were calculated using Student’s t-test (^*^ *P* < 0.05).

Clonogenic survival assays were performed to analyze the impact of adding vitamin C before or after RT on the colony-forming ability of different breast cancer cells. As expected, the level of radiosensitivity differed between the three cell lines, where SKBR3 exhibited the highest resistance to RT alone compared to MDA-MB-231 and MCF7. Compared to RT alone, vitamin C conferred non-significant radioresistance in MDA-MB231, MCF7, and to a lesser extent in SKBR3 after a combination of RT and vitamin C (Figure 1A); however, none of the survival fractions were significantly changed by vitamin C. Taken together, the results reveal that no statistically significant changes occurred after addition of vitamin C, either before or after of irradiation, compared to RT alone.

### The vitamin C concentration and combination treatment impacts cell proliferation in a cell line dependent manner

To ascertain whether the radioresistance effect of vitamin C was dependent on different concentrations of vitamin C and the sequence order of the treatments, MTT assays were performed after 72 h following 8 Gy of RT and either pre- or post-treatment of various vitamin C concentrations (Figure 1B).

There was a tendency towards radioresistance when the cells were treated with vitamin C before RT with the lower concentrations of vitamin C (0.12 and 0.25 mM), especially in SKBR3 and MDA-MB-231, compared to RT alone. Notably, with higher concentrations of vitamin C, the viability of the cells was dramatically impaired compared to RT alone, irrespective of whether vitamin C was given alone or before or after RT. However, higher pharmacological concentrations of vitamin C before RT were needed to significantly reduce the viability of SKBR3 and MDA-MB-231 cells, while higher pharmacological concentrations of vitamin C after RT were needed to significantly reduce the viability of MCF7 cells. Moreover, lower concentrations of vitamin C were needed to reduce the cell viability in SKBR3 cells, compared to MDA-MB-231 and MCF7, which illustrated that SKBR3 is more sensitive to the addition of vitamin C. Taken together, these findings imply that the sensitivity with respect to cell viability after the addition of vitamin C differs between the three irradiated cell lines.

### The addition of vitamin C conferred only minor alterations to the cell cycle arrest induced by RT alone

**Figure 2:**
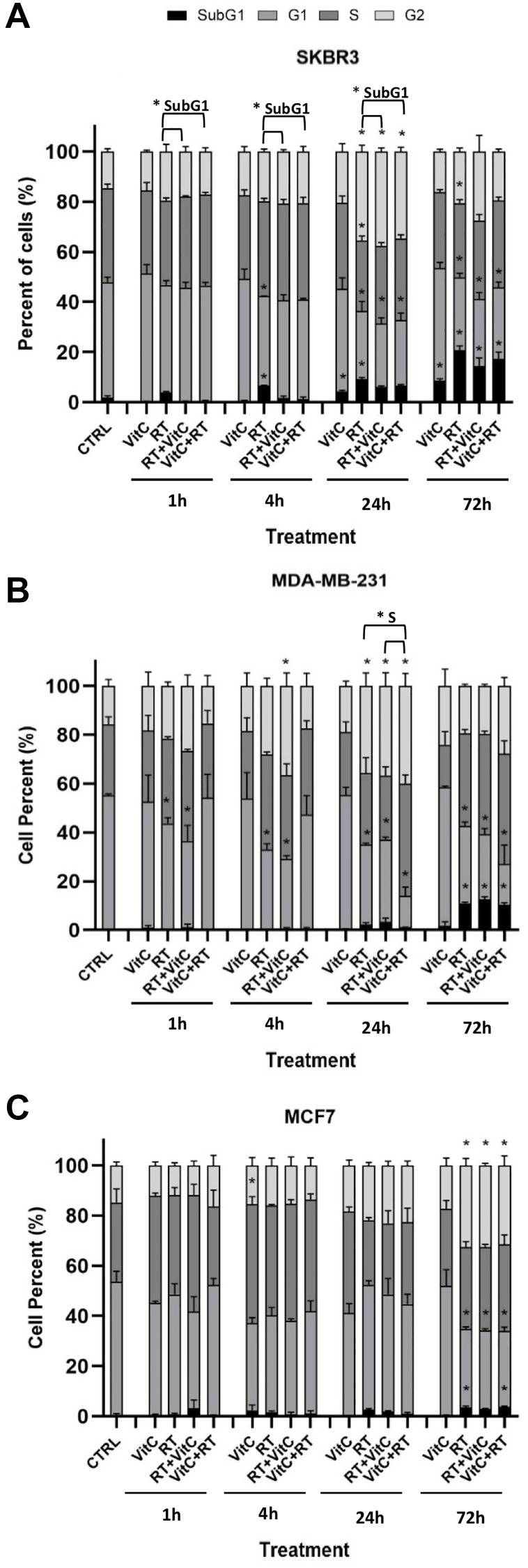
Effects of VitC and/or irradiation on cell cycle distribution in three breast cancer cell lines. Cellular DNA content of SKBR3 (**A**), MDA-MB-231 (**B**), and MCF7 (**C**) cells was determined by flow cytometry, and the distribution of cells was analyzed by FlowJo software. Results are presented as percentage ± SEM and are based on three independent experiments. Statistical analysis was carried out using one-way ANOVA followed by Tukey post-test at statistical significance *P* < 0.05 for treated cells compared to untreated control within each phase of the cell cycle for each time point. ^*^ *P* < 0.05 for VitC+RT or RT+VitC treated cells compared to RT alone within each phase of the cell cycle for each time point is also indicated.

Analyses of the cell cycle distribution by flow cytometry to investigate whether the inhibitory effects of vitamin C and RT were mediated through cell cycle arrest were done. The results showed a significant relative increase of the cell population in the G2 phase and a relative decrease in the G1 phase of the cell cycle after treatment with RT alone or in combination after 24 h in both SKBR3 (Figure 2A) and MDA-MB-231 cells (Figure 2B), and after 72 h in MCF7 cells (Figure 2C), compared to untreated control. Furthermore, after 72 h, the cell population that underwent apoptosis was significantly increased compared to untreated control, as shown by the increase of subG1 after 72 h in all the studied cell lines.

These results indicate that the inhibitory effect of RT resulted in G2 cell cycle arrest and apoptosis, while the addition of vitamin C did not significantly change the effect of RT at most of the time points. However, the combination treatment might delay the cell cycle arrest in different phases at some time points, depending on the cell type and whether cells were treated with vitamin C before or after RT as indicated.

### The addition of vitamin C conferred only minor alterations in the levels of several key proteins involved in radiation response, proliferation, or apoptosis

**Figure 3:**
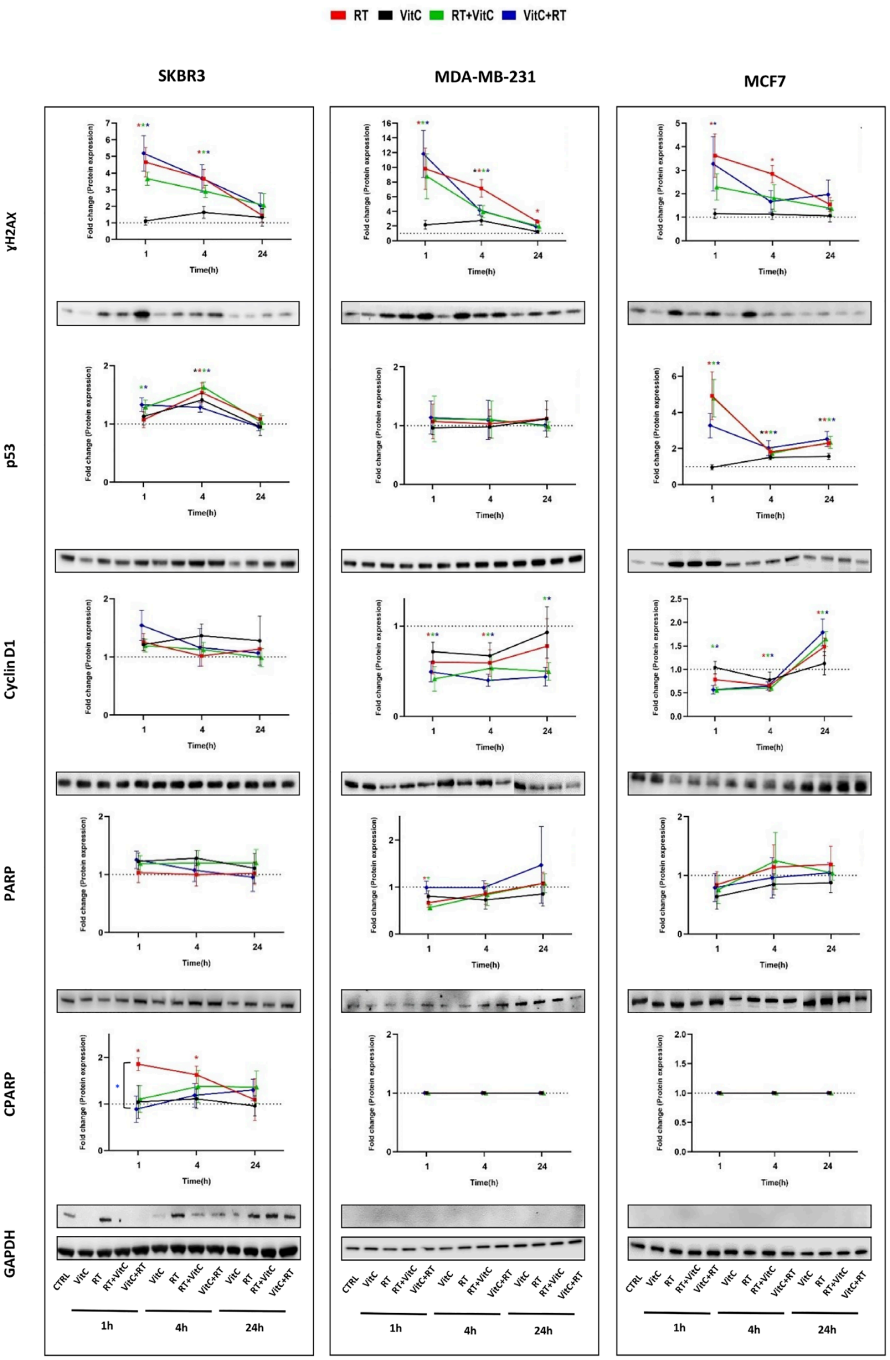
Protein expression of γH2AX, P53, CyclinD1, PARP, and cleaved PARP detected by Western blot in breast cancer cells treated with VitC and/or irradiation. The quantitative value of γH2AX, P53, CyclinD1, PARP, and cleaved PARP protein levels in SKBR3, MDA-MB-231, and MCF7 cells was assessed at various time points using whole cell lysates. GAPDH was used as an internal control to indicate the loading quantity. Each line corresponds to ± SEM of at least three independent experiments, and each band is a representative for each protein. Some gels have been cut between time points due to high number of samples. Statistical analysis was performed using the two-tailed Student’s t-test at statistical significance ^*^ *P* < 0.05 was considered statistically significantly different from untreated control. Differences between VitC+RT or RT+VitC treated cells and RT alone for each time point is also indicated.

Western blot assays were performed to analyze the expression of proteins involved in radiation response (γH2AX and p53), proliferation (Cyclin D1), and apoptosis (PARP, and cleaved PARP) in breast cancer cells treated with different combinations of RT and vitamin C as well as RT alone or vitamin C alone (Figure 3).

The γH2AX level increased equally after RT alone and pre- or post-treatment with vitamin C in SKBR3 cells and MDA-MB-231 cells, except MDA-MB-231 cells treated with RT alone after 24 h, compared to control. In MCF7, post-treatment with vitamin C resulted in a non-significantly lower level of γH2AX compared to RT alone, suggesting some radioprotection.

In SKBR3 cells, the p53 level increased only with the combination of vitamin C and RT after 1h and was equally increased in RT and pre- or post-treatment with vitamin C after 4 h. In MDA-MB-231 cells, the p53 level remained unchanged. In contrast, the p53 levels in MCF7 cells showed a significant increase at all studied time points and treatment conditions.

Cyclin D1 was not altered with the different treatments at any of the time points in SKBR3 cells. The results showed that in MDA-MB-231 cells, all treatments resulted in a decrease of Cyclin D1 levels. At 24 h, the Cyclin D1 level in RT only treated cells was similar to the control, while the Cyclin D1 level in pre- or post-treated cells was still significantly lower than the control. MCF7 cells showed a lower cyclin D1 level after pre- or post-treatment with vitamin C at 1 h compared to control. Cyclin D1 levels were similar after RT alone or in combination with vitamin C at the other time points.

Determination of PARP protein levels revealed that no significant changes occurred in different treatment conditions or time points in SKBR3 and MCF7 cells. However, the PARP levels were reduced after RT treatment alone or post-treatment with vitamin C in MDA-MB-231 cells after 1 h.

Cleaved PARP was significantly increased in SKBR3 cells treated with RT alone at 1 and 4 h compared to untreated control and was also significantly different between combination treatment and RT alone at 1 h. In MDA-MB-231 and MCF7 cells, no cleavage of PARP occurred compared to untreated control.

Taken together, no significant differences were observed between the cells that were treated with RT alone compared with pre- or post-treatment with vitamin C of irradiated cells except cleaved PARP in SKBR3 cells at 1 h.

### Supplement use in relation to patient and tumor characteristics

**Figure 4:**
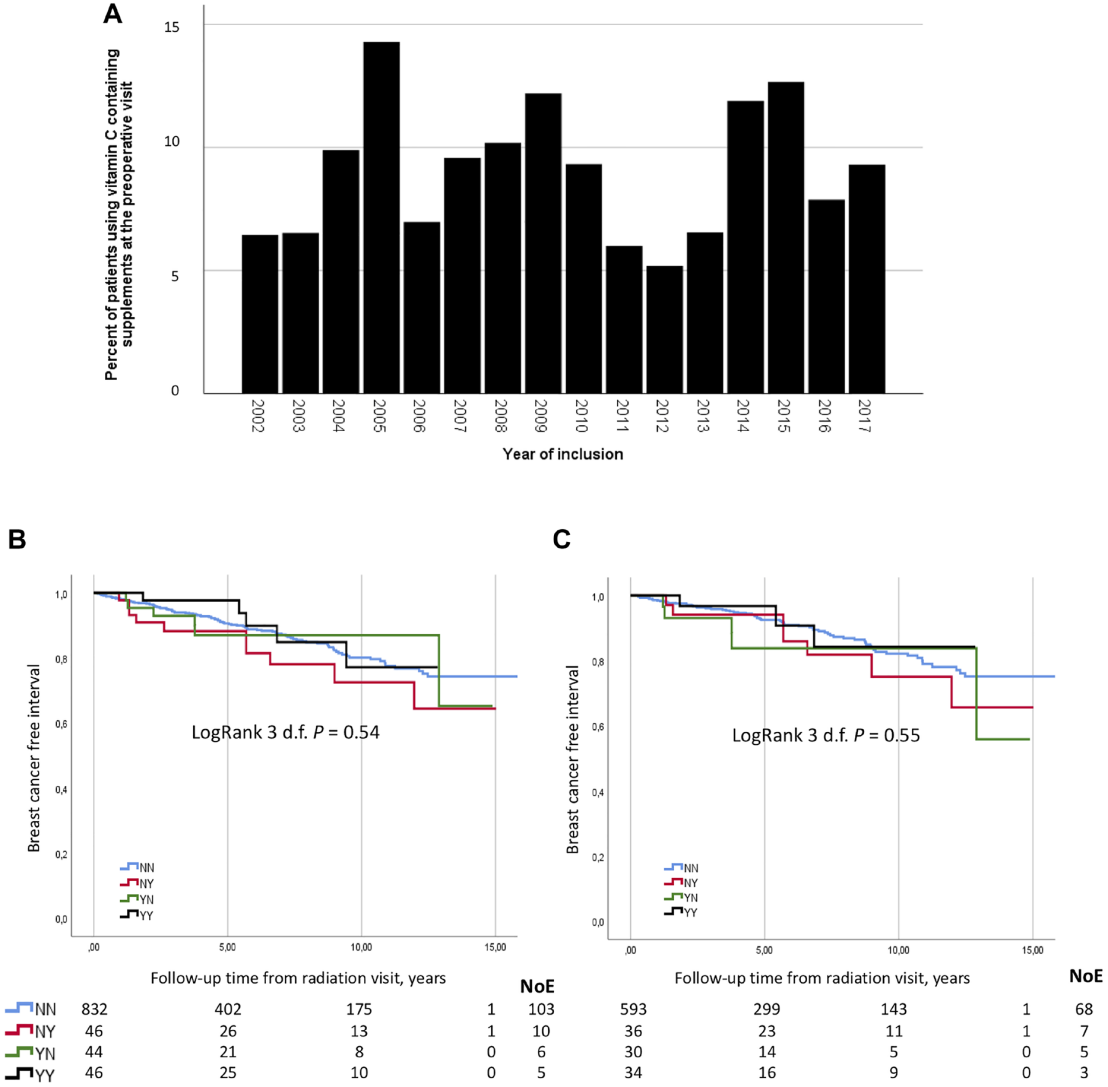
(**A**) The percentage of vitamin C use among primary breast cancer patients at the preoperative visit between 2002 and 2017. (**B**) Breast cancer free interval did not differ according to vitamin C use before and at the end of RT in 968 patients. (**C**) Breast cancer free interval did not differ according to vitamin C use before and at the end of RT in 693 chemonaïve patients.

Among the 1803 included patients, 163 (9.0%) reported vitamin C-containing supplements at inclusion. Between 2002 and 2017, vitamin C supplement use fluctuated (5.2–14.7%) and no trend was seen (Figure 4A). Of the 163 vitamin C users, 88 used multivitamins. Patients often reported the use of multiple supplements. Patient- and tumor characteristics in relation to vitamin C use are presented in Table 1. Patients using vitamin C supplements preoperatively were more likely to have a lower BMI < 25 kg/m^2^, to be nulliparous, and somewhat more likely to have used oral contraceptives, but were similar to non-users with respect to age, menopausal hormone therapy use, coffee consumption, alcohol abstention, and smoking status. Moreover, vitamin C users had tumors of lower histological grade and more often of lobular type compared with non-users. The association between vitamin C use and lower histological grade remained significant after adjustment for tumor size, ER and PR status, nodal involvement, age, and BMI. No association between preoperative vitamin C use and hormone receptor status or systemic treatments was observed.

**Table 1:** Patient and tumor characteristics at inclusion prior to surgery in relation to vitamin C containing supplement use

			Vitamin C at inclusion			Vitamin C at inclusion (Radiation therapy and follow-up visits)		
	All	Missing	No	Yes	*P*-value	No	Yes	*P*-value
	*n* = 1803		*n* = 1640	*n* = 163		*n* = 887	*n* = 81	
	Number (%)		Number (%)	Number (%)		Number (%)	Number (%)	
Age ≥50 years	1452 (80.5)	0	1321 (80.5)	131 (80.4)	>0.3	702 (79.1)	68 (84.0)	>0.3
BMI ≥25 kg/m^2^	883 (52.1)	107	819 (53.2)	64 (40.8)	0.003	471 (55.8)	32 (41.0)	0.012
Parous	1592 (88.3)	1	1456 (88.8)	136 (83.4)	0.040	802 (90.4)	67 (82.7)	0.029
Oral contraceptive use, ever	1328 (73.8)	3	1198 (73.2)	130 (79.8)	0.069	661 (74.6)	63 (77.8)	>0.3
Menopausal hormone therapy, ever	714 (39.8)	7	641 (39.2)	73 (45.1)	0.15	354 (40.0)	41 (50.6)	>0.3
Coffee ≥2 cups/day	1435 (79.6)	0	1310 (79.9)	125 (76.7)	>0.3	713 (80.4)	65 (80.2)	>0.3
Alcohol abstention	213 (11.9)	8	193 (11.8)	20 (12.3)	>0.3	95 (10.7)	8 (9.9)	>0.3
Current smoker	314 (17.5)	9	282 (17.3)	32 (19.6)	>0.3	167 (18.9)	15 (18.5)	>0.3
**Invasive tumor size**								
>20 mm or skin or muscular involvement	461 (25.6)	0	424 (25.9)	37 (22.7)	>0.3	196 (22.1)	19 (23.5)	>0.3
**Any axillary lymph node** **involvement, yes**	600 (33.3)	2	540 (33.0)	60 (36.8)	>0.3	321 (36.2)	33 (40.7)	>0.3
**Histological grade III, yes**	491 (27.3)	6	462 (28.3)	29 (17.8)	0.004	232 (26.2)	10 (12.3)	0.006
**Main histological type**		0			0.017			0.006
No special type (formerly ductal)	1438 (79.8)		1316 (80.2)	122 (74.8)		718 (80.9)	62 (76.5)	
Lobular	211 (11.7)		181 (11.0)	30 (18.4)		95 (10.7)	17 (21.0)	
Other or mixed	154 (8.5)		143 (8.7)	11 (6.7)		74 (8.4)	2 (2.5)	
**Receptor status**								
ER^+^	1593 (88.6)	6	1447 (88.5)	146 (90.1)	>0.3	785 (88.7)	75 (92.6)	0.28
PR^+^	1285 (71.5)	6	1176 (71.9)	109 (67.3)	0.21	646 (73.0)	57 (70.4)	>0.3
HER2^+^	176 (10.1)	68	164 (10.4)	12 (7.7)	0.29	93 (10.8)	5 (6.5)	0.23
Triple negative	138 (7.7)	12	126 (7.7)	12 (7.4)	>0.3	66 (7.5)	5 (6.2)	>0.3
**Systemic therapy prior** **to last follow-up** or any recurrence^*^								
Tamoxifen	919 (51.0)	0	839 (51.2)	80 (49.1)	>0.3	455 (51.3)	43 (53.1)	>0.3
Aromatase inhibitor	743 (41.2)	0	674 (41.1)	69 (42.3)	>0.3	375 (42.3)	37 (45.7)	>0.3
Chemotherapy	516 (28.6)	0	478 (29.1)	38 (23.3)	0.12	258 (29.1)	17 (21.0)	0.12
Trastuzumab	127 (7.0)	0	120 (7.3)	7 (4.3)	0.15	66 (7.4)	1 (1.2)	0.035

ER Estrogen Receptor, PR Progesterone Receptor, HER2 Human Epithelial Growth Factor Receptor-2 ^*^Patients may have received more than one type of postoperative therapy.

### Vitamin C and recurrence-free interval in RT treated patients

There were 968 patients treated with RT who provided information on vitamin C supplement intake at the follow-up visit by the end of RT. By June 30, 2019, 796 were alive and recurrence-free, with a median follow-up time as of the RT visit of 4.9 years (IQR 2.7–8.8). The median time-point of the follow-up visit in relation to the end of RT was 1 day before the end of RT (IQR 3 days before the end of treatment to 5 days after the end of RT). There was a total of 124 breast cancer recurrences, of which 30 were loco-regional. There was no difference in the recurrence-free interval in the univariable (LogRank 3 d.f. *P* = 0.54; Figure 4B) or multivariable model (3 d.f. *P* = 0.76), adjusted for clinicopathological factors, smoking, and adjuvant treatments. Similar there was no difference for loco-regional recurrence-free interval (LogRank 3 d.f. *P* = 0.88) according to vitamin C status at the two visits (NN, NY, YN, and YY).

Among the 968 patients, 693 were chemonaïve and had 83 breast cancer recurrences (23 loco-regional). There was no difference in the recurrence-free interval in the univariable (LogRank 3 d.f. *P* = 0.55; Figure 4C) or multivariable model (3 d.f. *P* = 0.65). Again there was no difference for loco-regional recurrence-free interval (LogRank 3 d.f. *P* = 0.76) according to vitamin C status. A restriction analysis including 586 patients (80 recurrences, 23 loco-regional) for whom the follow-up took place during or a maximum of 1 week after the end of RT showed no difference in recurrence-risk according to vitamin C status.

## DISCUSSION

The current study demonstrated that the addition of maximally orally obtainable doses (0.22 mM) of vitamin C before or after radiation treatment (RT) of breast cancer cells resulted only in non-significant increases in colony formation. In addition, pharmacological concentrations of vitamin C (0.5–20 mM) alone or in combination with RT resulted in reduced cell proliferation in MTT assays, while pretreatment of RT cells with 0.22 mM of vitamin C slightly increased proliferation in all three cell lines. However, treatment with a combination of RT and vitamin C induced only minor differences in the cell cycle phases arrest or expression of proteins involved in DNA damage repair, cell cycle regulation, and apoptosis, compared to RT alone. Preoperative use of supplements with vitamin C in breast cancer patients fluctuated over time and was associated with lower BMI, nulliparity, lower histological grade, and lobular breast cancer. Despite less aggressive tumors, there was no difference in recurrences in RT-treated patients according to vitamin C status.

To our knowledge, this is the first time an interaction between the maximum dose of vitamin C achievable from oral administration and RT has been investigated in human breast cancer cells *in vitro*. Other *in vitro* studies in other cancer types, such as leukemia, primary glioblastoma, epithelial cancer, and sarcoma, have shown radio-sensitizing effects of pharmacological doses of vitamin C combined with irradiation [19, 21, 27].

Radioresistance measured by clonogenic survival assay with pre- or post-treatment with vitamin C in irradiated breast cancer cells was not dependent on the treatment order but slightly cell line dependent. Furthermore, the cell viability assay (MTT) showed that higher doses of pre-or post-treatment with vitamin C were needed in MCF7 and MDA-MB-231 than in SKBR3 to alter the treatment effect compared to RT alone, indicating that SKBR3 was the most sensitive of these cell lines to vitamin C. The results align with a previous study that implied that the differential vitamin C sensitivity was related to the sodium-dependent vitamin C transporter2 (SVCT-2) protein levels. These levels are lower in MDA-MB-231 and higher in SKBR3 [26]. The results of the MTT assay also suggest that the cell sensitivity was dependent on the vitamin C concentration and, to a lesser extent, the treatment order. The highest physiological concentrations, which can be obtained from oral administration of vitamin C [14], might increase cell viability when added before RT. However, pharmacological concentrations of vitamin C, which can only be obtained through intravenous administration of vitamin C [14], were needed to reduce the cell viability when vitamin C was added alone or as pre- or post-treatment of RT cells. These findings suggest that lower doses of vitamin C combined with RT lead to higher radioresistance than higher doses of vitamin C. The MTT results also suggest that vitamin C in doses seen in supplements increase cancer cell proliferation.

The cell cycle phases were similar in response to RT alone or the combination of RT and vitamin C. Recent studies indicated that physiological doses of vitamin C could induce apoptosis and inhibit metastatic activity in breast cancer cell lines [28, 29]. In the current study, vitamin C alone induced apoptosis only in SKBR3, as exhibited by the increasing SubG1phase arrest in this cell line.

Moreover, γH2AX, one of the most sensitive markers of radiation-induced DNA damage and radiation response [30, 31], p53, which play a role in response to cellular stresses, including DNA damage and cell cycle regulation [32, 33], and cyclin D1, an important regulator of cell cycle and proliferation [34], showed no significant differences in cells treated with a combination of RT and vitamin C, compared to RT alone. Cleaved PARP, which is a hallmark of apoptosis [35], differed only in SKBR3 depending on when vitamin C was added to RT, compared to RT alone at 1 h.

Numerous studies have demonstrated a cytotoxic effect of vitamin C at concentrations of 1 mM or above on tumor cells *in vitro*. The cytotoxicity in many of these studies reflects the oxidative stress resulting from the H_2_O_2_ generated when vitamin C is present [36]. The current study suggests that vitamin C combined with irradiation could not statistically significantly alter the colony formation, cell viability, cell cycle arrest, and regulation of the investigated proteins. However, there was a tendency towards slightly lower radio efficiency in all the cell lines with combination treatment.

The clinical data showed no significant difference in recurrence-risk according to vitamin C status. Post diagnosis vitamin C use has previously been associated with a better prognosis overall [37, 38], but not in RT-treated patients [38]. Further, post-operative vitamin C use was not associated with breast cancer-specific- or total mortality in a previous Swedish breast cancer cohort [39], in line with our results.

An association between supplement use and a lower BMI is similar to other studies in breast cancer patients [1, 37, 40]. Campbell *et al*. showed that higher levels of ascorbic acid in the DNA of breast cancer were associated with a lower tumor grade, in line with our results. However, there was no association between ascorbic acid levels in normal tissue compared with tumor tissue [41]. Differences in vitamin C uptake between normal and neoplastic tissue have been observed previously [42], suggesting that patient intake might not fully reflect the capability of the tumor to utilize the vitamin C.

The estimates of the use of vitamin C in this study have some methodological limitations. Most patients combined their vitamin C intake with other supplements or used combination supplements, similar to other studies [37, 39]. This fact limits the conclusions that can be drawn on the role of vitamin C alone. At the follow-up visits, patients reported supplement use during the past week, which reduces the risk of recall bias. However, supplements may not have been taken close to the daily RT treatment. Although the total number of patients was large, the number of RT-treated vitamin C users was smaller. RT-treated patients with complete vitamin C data were similar to all patients, suggesting that patients included in survival analyses were representative for this population-based cohort.

In the *in vitro* experiments, we choose the highest concentration of vitamin C obtainable by oral administration since vitamin C in culture conditions is fairly unstable due to oxygen exposure and temperature. However, lower doses may have better represented plasma concentrations after vitamin C supplement use. Preliminary experiments with lower vitamin C concentrations (15, 30, and 60 μM) revealed no significant differences when compared to 120 and 250 μM of vitamin C (data not shown).

In conclusion, vitamin C impacted RT efficiency differently depending on breast cancer subtype and vitamin C concentration. Lower doses of vitamin C, such as those achievable with oral administration, might increase breast cancer cell proliferation and decrease radiosensitivity. Despite vitamin C users having less aggressive tumors than non-users, the recurrence risk in RT-treated patients was similar in vitamin C users and non-users.

## MATERIALS AND METHODS

### Cell lines, cell culture, and treatments

The human breast cancer cell lines SKBR3 (ATCC-HTB-30; Lot No:59510062), MDA-MB-231 (ATCC-HTB-26; Lot No:70006554), and MCF-7 (ATCC-HTB-22; Lot No:70019550) were purchased from the American Type Culture Collection (ATCC, Manassas, USA). SKBR3 cells were maintained in McCoy (HyClone, Chicago, USA), MDA-MB-231 cells were cultured in Dulbecco’s modified Eagle’s medium (Gibco, Gaithersburg, USA), and MCF7 cells were grown in Roswell Park Memorial Institute (RPMI 1640 (Gibco). All media were supplemented with 10% FBS (Sigma Aldrich, Missouri, USA) and penicillin-streptomycin (1%) (Gibco). The cells were cultured for no more than 20 passages and maintained at 37°C in a humidified incubator containing 5% CO_2_. Cells were checked and confirmed to be negative for mycoplasma contamination. To induce an ionizing radiation response, the cells were irradiated with a 225 kVp X-ray source (Xstrahl Ltd., Brownhills West Midlands, UK) at the division of Medical radiation physics, Lund University, Lund, Sweden.

For vitamin C treatment, the cells were exposed to vitamin C (L-Ascorbic acid) (Sigma Aldrich) in the dark at 37°C for 1 h followed by washing with phosphate-buffered saline (PBS; HyClone, Chicago, USA) either before or after irradiation. Since starving cells prior to irradiation may affect the radiosensitivity and interfere with different pathways, such as p53 [43, 44], serum-free media was not used in order to mimic physiological conditions.

### Colony formation assay

Exponentially growing cells were plated at different densities in 25 cm^2^ flasks, due to different plate efficiencies for the three cell lines. After 24 h, the cells were irradiated with 2, 4, 6, or 8 Gy before or immediately after 1 h treatment with 0.22 mM vitamin C. Cells were incubated for 14 days, and the medium was changed every 3 days. Colonies were fixed and stained with a 0.3% methylene-blue in ethanol, and the flasks scanned using an Epson V800 scanner. Only colonies containing more than 50 cells were counted as surviving colonies using ImageJ software (NIH, USA). The plating efficiency (PE) was determined for non-irradiated flasks, with or without vitamin C. Survival fraction (SF) was calculated as follows:


$$\text{SF}=\frac{\text{colonies counted}}{\left(\text{cells seeded }\times \;{\text{PE}}_{\text{own control}}\right)}$$

All assays were independently performed in three sets of experiments in triplicate samples.

### Cell viability analysis

The cells were plated at a density of 5 × 10^3^ cells/well for 24 h followed by treatment with various concentrations of vitamin C (0.125, 0.25, 0.5, 1, 2.5, 5, 10, and 20 mM) before or after 8 Gy irradiation. After 72 h, 1 mg/ml 3-(4,5-dimethylthiazol-2-yl)-2,5-diphenyltetrazolium bromide (MTT, Sigma Aldrich) was added to the wells and the plate incubated for 3 h. The MTT solution was removed, and dimethyl sulfoxide (DMSO; Sigma Aldrich) was added to each well to dissolve the formazan crystals by shaking the culture plates. Following incubation at room temperature for 15 min, the optical densities of the formazan solution in each well were measured at 590 nm using FLUOstar Omega microplate reader (BMG Labtech, Ortenberg, Germany). Wells with no treatment were considered as the control, and cell viability percentage was calculated as:


$$\text{Cell viability percentage}=\frac{\text{Absorbance of treated cells}}{\text{Absorbance of control }\times \;100}$$

### Cell cycle distribution analysis

Cells were seeded into 25 cm^2^ culture flasks at a density of 5 × 10^5^ cells/ml and allowed to adhere overnight, followed by treatment with 0.22 mM vitamin C before or after 8 Gy irradiation. After 1, 4, 24, and 72 h of treatment, the cells were washed with cold PBS, re-suspended in 500 μl PBS, and fixed in 70% cold ethanol for at least 2 h in −20ºC. The cells were then washed with PBS twice, followed by incubation with a mixture of 500 μl PI/RNase (400 μl propidium iodide and 100 μl Ribonuclease A). Stained cells were incubated at room temperature in the dark for 30 min before analysis. The cell cycle profile was determined by using *BD* Accuri^®^ *C6* (Becton Dickinson, NJ, USA), and the percentage of cells in each phase of the cell cycle was quantified by fitting the histograms with the Watson model in the FlowJo version 10.2 software. Three independent experiments were carried out, and the data were expressed as the percentage of cells compared to the untreated control or RT alone.

### Protein extraction and Western blot

Cells were seeded at a 5 × 10^5^ cells/ml density in 25 cm^2^ and irradiated before or after treatment with 0.22 mM vitamin C. The treated cells were harvested at indicated time points (1, 4, and 24 h). The cells were washed with cold PBS and centrifuged at 3000 rpm at 4°C for 5 min. The centrifuged cells were resuspended with Radioimmunoprecipitation assay (RIPA) lysis buffer containing 50 mM Tris-HCl, pH 8.0, with 150 mM sodium chloride, 1.0% Igepal CA-630 (NP-40), 0.5% sodium deoxycholate and 0.1% sodium dodecyl sulfate (Sigma Aldrich) with additional protease and phosphatase inhibitor cocktail (Roche, Basel, Switzerland). The lysates were incubated on ice for 20 min and then centrifuged at 13,000 rpm and 4°C for 20 min.

The concentrations of the protein samples were determined using Pierce BCA Protein Assay Kit (Pierce Biotechnology, Massachusetts, USA) at 540 nm using FLUOstar Omega microplate reader. Equal quantities of protein were separated by SDS-PAGE (4–12% Bis-Tris-Protean gel, Invitrogen) and transferred to a nitrocellulose membrane (Trans-Blot Turbo™ mini Nitrocellulose Transfer Packs, Bio-Rad). The membrane was blocked with 5% bovine serum albumin (BSA) (Sigma Aldrich) in Tris-buffered saline buffer containing 0.1% Tween-20 (TBS/T). The following antibodies were used: rabbit monoclonal anti-cyclin D1 antibody (M3642, Dako, Glostrup, Denmark); rabbit Polyclonal anti-PARP antibody (9542, Cell Signaling Technology, Massachusetts, USA) and anti-cleaved-PARP antibody (9541, Cell Signaling Technology); mouse monoclonal anti- P53 antibody (DO-1, SC-126, Santa Cruz Biotechnology, Texas, USA), anti-γH2AX antibody (AJ1351a, Nordic biosite, Täby, Sweden), and anti-GAPDH antibody (MAB374, Sigma). The blots were developed using horseradish peroxidase-conjugated secondary antibody. SuperSignal™ West Dura Extended Duration Substrate (Thermofisher Scientific) was used to detect protein bands with Odyssey imaging system (LI-COR Biosciences, Nebraska, USA). Densitometry analysis was performed using Image studio version 5.2 software, and the densities were normalized using GAPDH as the internal control. Some gels have been cut between time points due to high number of samples.

### Study population

**Figure 5:**
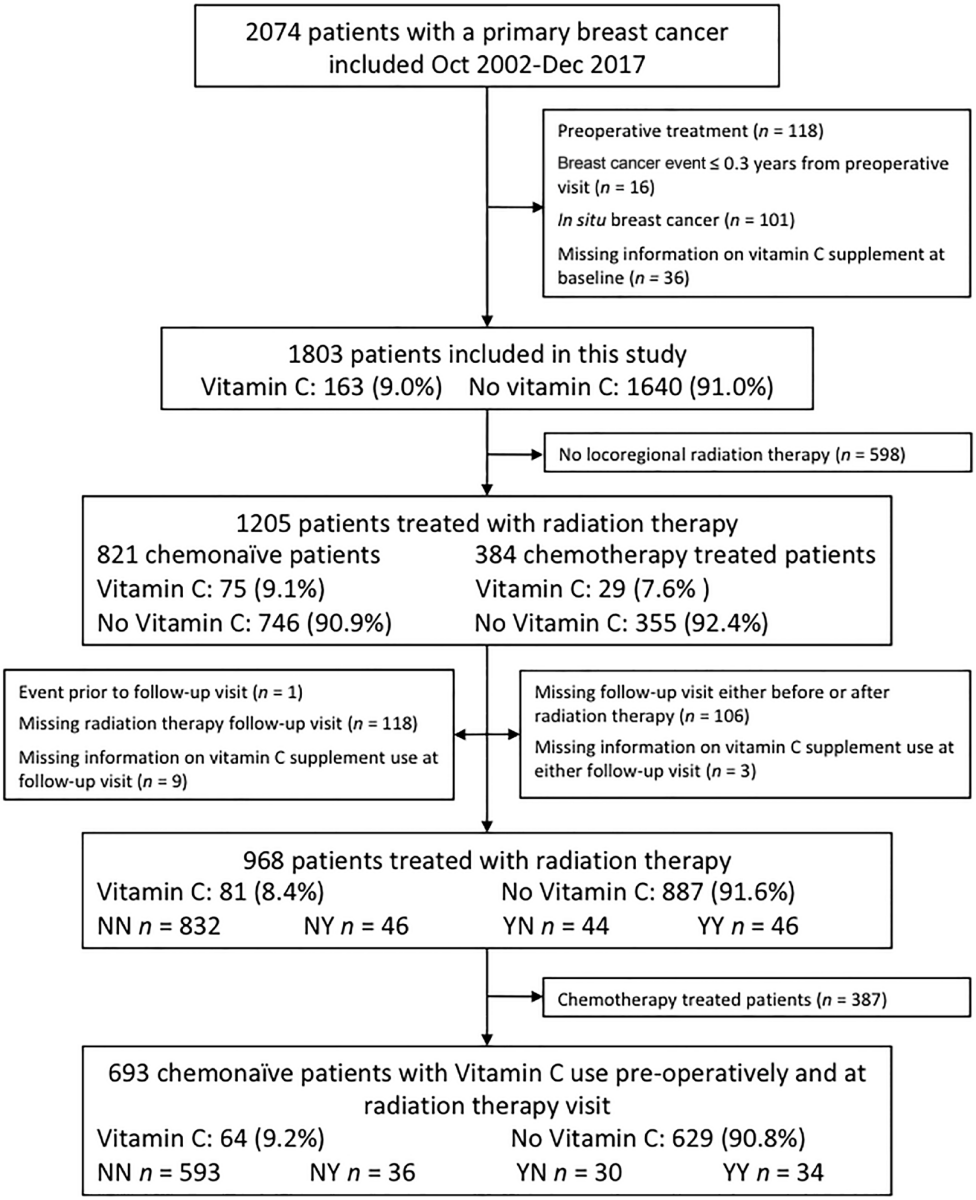
Inclusion flowchart showing included and excluded patients for survival analysis with regard to the use of supplements containing vitamin C.

Clinical data from 1803 patients with primary invasive breast cancer who had not received preoperative treatment and had available data on vitamin C use were included in the BC-blood study 2002–2017 in Lund, Sweden. Details of the cohort have been previously published [45]. In brief, at the preoperative visit, the patients completed a 3-page questionnaire with information on reproductive factors, lifestyle, medication and supplement use during the past week, and food intake on the day of study inclusion. Body measurements were obtained by a trained research nurse, and the body mass index (BMI) kg/m^2^ was calculated. Follow-up visits took place during or after the completion of chemotherapy (if applicable) and RT. Of the 1803 included patients, 968 patients had received RT and completed questionnaires before and after RT. Of these, 693 were chemonaïve, see flowchart (Figure 5). Ethical approval was obtained from the Lund University Ethics Committee (Dnr75-02, Dnr37-08, and Dnr658-09 with amendments) and written informed consent was obtained from all participants.

Tumor characteristics, including tumor size, axillary lymph node involvement, ER and progesterone receptor (PR) status, and histological type and grade, were retrieved from pathology reports [45]. HER2 status was obtained from pathology reports or from dual gene protein staining of tumor microarrays [46]. In case of bilateral invasive cancer (*n* = 30) data from the most aggressive tumor is presented. Adjuvant treatments were recorded until any first breast cancer event, death, or last follow-up. Breast cancer events were gathered from patient charts and the Regional Tumor Registry. The date of death was retrieved from the Swedish Population Registry. The last update of the cohort was June 30, 2019.

Information on vitamin C content in supplements was retrieved from the internet and product home pages. Supplements reported as “multivitamins” were considered to include vitamin C since most Swedish multivitamins include vitamin C [47] as well as botanical supplements with added vitamin C. Due to difficulty in assessing the vitamin C content in pure botanical supplements, these supplements were coded as not including vitamin C.

### Statistical analysis

The statistical software packages SPSS version 26 (IBM Corp, Armonk, NY, USA) and GraphPad version 8 (San Diego, California, USA) were used. Data for the mechanistic parts were presented as mean values with standard error of the mean (±SEM) and were analyzed using the two-tailed Students *t*-test for comparisons between two groups. One-way ANOVA with Tukey’s post hoc test was used for comparisons between multiple groups.

For the clinical data, clinicopathological features at the inclusion during the preoperative visit was compared between vitamin C users and non-users. The following variables were dichotomized: age at inclusion (≥50 years), BMI (≥25 kg/m^2^) and current smoker (yes), tumor size (>20 mm or skin or muscular involvement), any axillary lymph node involvement (yes), histological grade III (yes), ER^+^, PR^+^, or HER2^+^ (yes), and triple-negative (yes). Chi-square and logistic regression were used to assess vitamin C use in relation to clinicopathological features in univariable and multivariable models. Patients reporting supplement use before surgery or at the follow-up visit were considered users at that time point. Use of vitamin C was categorized in relation to preoperative use or at the end of chemotherapy (if applicable) yes (Y)/no (N) and use at the RT follow-up visit, yielding four different categories: YY, YN, NY, and NN [2]. In a restriction analysis, only patients who completed the follow-up visit during or a maximum of 1 week after completing RT were included. Patients were followed until June 30, 2019, and survival analysis was conducted with Kaplan-Meier estimates with landmark analyses starting at the time of the follow-up visit. Cox proportional regression analysis was used for multivariable models adjusted for age, tumor size, node status, grade III, ER status, BMI, smoking, and adjuvant treatments. The recurrence-free interval for the endpoints ‘any loco-regional recurrence’ and ‘any first recurrence’ was calculated, and the follow-up time was censored at the date of last follow-up or death by June 30, 2019. All *P*-values were 2-sided, and *P*-values <0.05 were considered statistically significant.
